# Commentary: Genome Sequence of *Vibrio parahaemolyticus* VP152 Strain Isolated From *Penaeus indicus* in Malaysia

**DOI:** 10.3389/fmicb.2018.00865

**Published:** 2018-05-01

**Authors:** Theodore Allnutt, Chrystine Zou Yi Yan, Tamsyn M. Crowley, Han Ming Gan

**Affiliations:** ^1^Bioinformatics Core Research Group, Deakin University, Geelong, VIC, Australia; ^2^School of Medicine, Centre for Molecular and Medical Research, Deakin University, Geelong, VIC, Australia; ^3^School of Science, Monash University Malaysia, Petaling Jaya, Malaysia; ^4^Genomics Facility, Tropical and Biology Multidisciplinary Platform, Monash University Malaysia, Petaling Jaya, Malaysia; ^5^Poultry Hub Australia, University of New England, Armidale, NSW, Australia; ^6^Centre for Integrative, School of Life and Environmental Sciences, Deakin University, Geelong, VIC, Australia

**Keywords:** *Vibrio parahaemolyticus*, taxonomy, genomics, shrimp, South East Asia

*Vibrio parahaemolyticus* is a marine gram negative bacterium that has been gaining significant attention in the shrimp aquaculture industry given its direct association with early mortality syndrome (EMS) or acute hepatopancreatic necrosis disease (AHPND) in shrimps (Soto-Rodriguez et al., 2015). Despite its significant threat to the industry, the genomic representation of shrimp-associated *V. parahaemolyticus* isolated from Malaysia or South East Asia in general is relatively low (Kondo et al., 2014; Yang et al., 2014; Foo et al., 2017). Letchumanan and colleagues recently reported the draft genome of *V. parahaemolyticus* VP152 isolated from a banana prawn in Malaysia (Letchumanan et al., 2016b). Strain VP152 was sequenced on the Illumina MiSeq and its whole genome sequence was deposited in DDBJ/EMBL/GenBank under the accession number and Bioproject ID of LCUL01000000 and PRJNA281142, respectively.

The G+C content for strain VP152 was reported to be 53.4% which is substantially higher than the average G+C content of *V. parahaemolyticus* (~45%) (Kondo et al., 2014; Yang et al., 2014; Foo et al., 2017). A similarity search of house-keeping genes coded in the genome of strain VP152 showed best hits to members of the genus *Citrobacter* (data not shown). A subsequent phylogenomic analysis using PhyloPhlAN (Segata et al., 2013) clustered strain VP152 with members of the genus *Citrobacter* with strong SH-like local branch support (Figure 1A). In addition, similar to several *Citrobacter* strains, when searched against the complete genome of *V. parahaemolyticus* ATCC 17802^T^, strain VP152 exhibited only modest genomic region with significant nucleotide homology to the *V. parahaemolyticus* reference genome (Figure 1B) (Alikhan et al., 2011). It is also worth noting that *V. parahaemolyticus* strain VP103 deposited in DDBJ/EMBL/GenBank under the accession number LBDB01000000 reported by the same group in a different data report (Letchumanan et al., 2016a) also showed the same phylogenomic affiliation to the genus *Citrobacter* instead of *Vibrio*.

**Figure 1:**
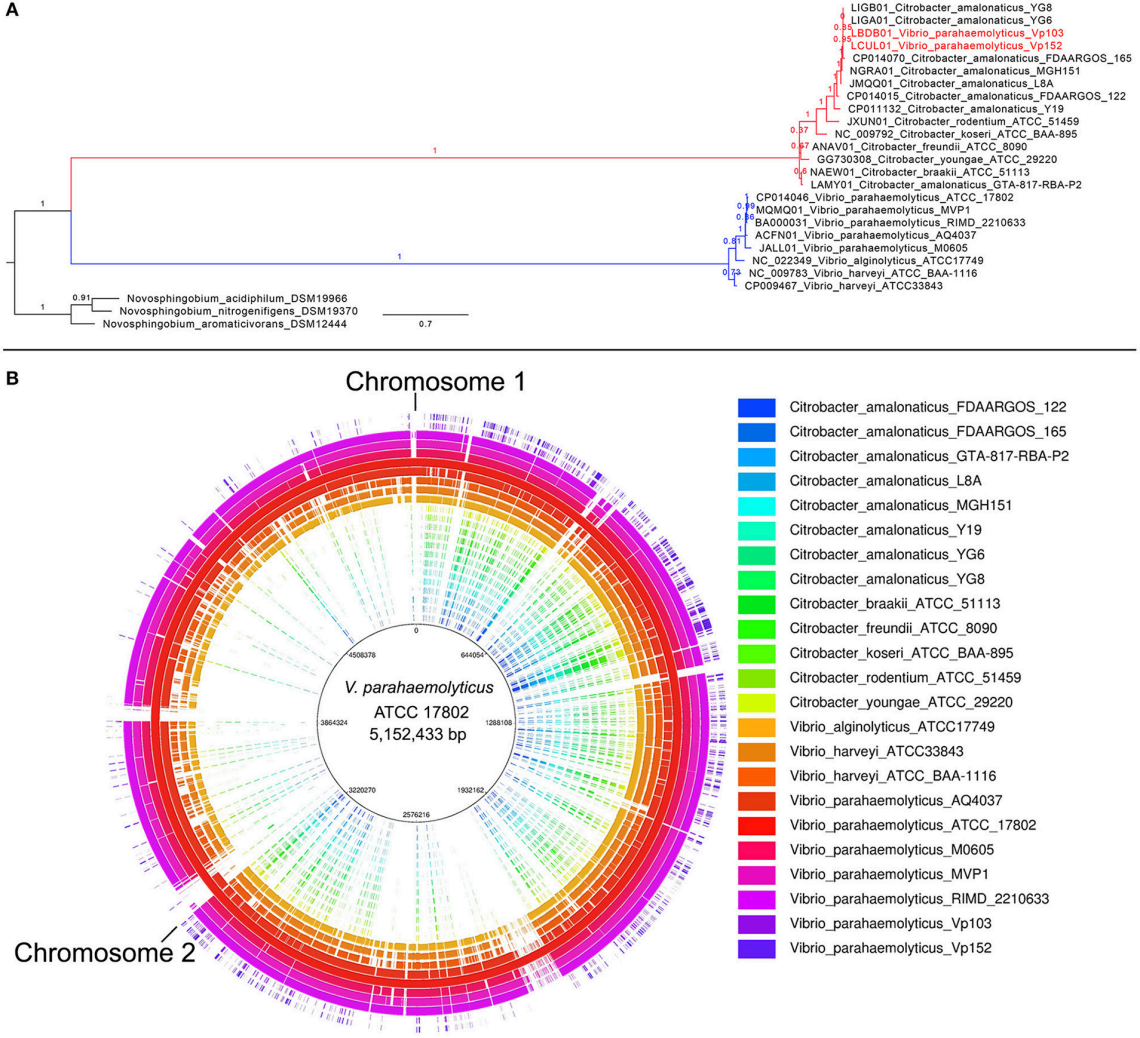
Potential misidentification of strain VP152 to the genus *Vibrio* as revealed by phylogenomic analysis and whole genome nucleotide comparison with *V. parahaemolyticus* ATCC 17802^T^. **(A)** Maximum likelihood tree constructed from the concatenated alignment of 400 universal proteins. The tree was rooted with members of the genus *Novosphingobium* as the outgroup. Node values indicate SH-like local branch support as implemented in FastTree2 and scale bar indicates the number of amino acid changes per site. **(B)** Whole genome comparisons of various *Vibrio* and *Citrobacter* strains against the complete genome of *V. parahaemolyticus* ATCC 17802^T^. Regions exhibiting significant homology (BLASTN with an *E*-value cut-off of 1E^−7^) were colored based on strain label.

Furthermore, a search in the NCBI bioproject database revealed that *C. amalonaticus* YG6 and *C. amalonaticus* YG8 with the Bioproject IDs of PRJNA292629 and PRJNA292637, respectively, were also sequenced by the same institute. This observation in addition to the monophyletic clustering of strains VP103 and VP152 with the two *Citrobacter* strains suggest potential sample mislabeling or barcode index misassignment during library preparation or sequencing.

Unfortunately, the authors did not describe any methodology associated with genome-based *in-silico* bacterial species validation in the data report to allow us to reproduce the identification of strain VP152 to the species *V. parahaemolyticus*. Given that the genome analysis of *V. parahaemolyticus* strain VP152 was based on the genome of a distantly related genus e.g. *Citrobacter*, it is unlikely that the biology interpretation in addition to the genome sequence reported in this study will be useful to the genomic study of *V. parahaemolyticus* or more generally the genus *Vibrio*.

## Author contributions

HG, TA, TC, and CY performed data analysis. HG wrote the manuscript. All authors proofread the manuscript.

### Conflict of interest statement

The authors declare that the research was conducted in the absence of any commercial or financial relationships that could be construed as a potential conflict of interest.
